# Long-term effects on growth of an energy-enhanced parenteral nutrition in preterm newborn: A quasi-experimental study

**DOI:** 10.1371/journal.pone.0235540

**Published:** 2020-07-06

**Authors:** Gianluca Terrin, Alessandra Coscia, Giovanni Boscarino, Francesca Faccioli, Maria Di Chiara, Carla Greco, Elisa Onestà, Salvatore Oliva, Marina Aloi, Lucia Dito, Viviana Cardilli, Daniela Regoli, Mario De Curtis

**Affiliations:** 1 Department of Maternal and Child Health Policlinico Umberto I, University La Sapienza, Rome, Italy; 2 Neonatology Unit, Department of Public Health and Pediatrics, University of Turin, Turin, Italy; Addis Ababa University School of Public Health, ETHIOPIA

## Abstract

**Aim:**

To assess the best energy intake in Parenteral Nutrition (PN) for preterm newborns, considering both possible benefits for growth and risk of complications.

**Methods:**

Quasi-experimental study comparing two cohorts of newborns, receiving Energy-Enhanced vs. Standard PN (Cohort A, from 1st January 2015 to 31 January 2016 and Cohort B from 1st February 2016 to 31 March 2017; respectively) after implementation of a change in the PN protocol. The primary outcome measure was growth at 24 months of life. The PN associated complications were also measured.

**Results:**

We enrolled 132 newborns in two Cohorts, similar for prenatal and postnatal clinical characteristics. Although, body weight and length at 24 months of life were significantly higher (p<0.05) in the Cohort A (11.1, 95% CI 10.6 to 11.6 Kg; 85.0 95% CI 83.8 to 86.2 cm) compared with Cohort B (10.4, 95% CI 9.9 to 10.9 Kg; 81.3 95% CI 79.7 to 82.8 cm), body weight and length Z-Score in the first 24 months of life were similar between the two Cohorts. The rate of PN associated complications was very high in both study Cohorts (up to 98% of enrolments). Multivariate analysis showed that length at 24 months was significantly associated with receiving standard PN (cohort A) in the first week of life and on the energy intake in the first week of life. We also found a marginally insignificant association between Cohort A assignment and body weight at 24 months of life (*p* = 0.060).

**Conclusions:**

Energy-enhanced PN in early life has not significant effects on long-term growth in preterm newborns. The high prevalence of PN associated complications, poses concerns about the utility of high energy intake recommended by current guidelines for PN.

## Introduction

Growth failure has been reported among up to 90% of preterm newborns at discharge from the neonatal intensive care unit (NICU) and it may persist in the first years of life [1,2]. The post-natal growth retardation remains a challenge for neonatologists. Adequate nutritional support immediately after birth is essential for preterm newborns to limit growth retardation [3,4]. Over the last decades, the early “aggressive” nutrition has been adopted in NICU to achieve a neonatal growth as close as possible to that of a fetus of the same gestational age (GA). Early aggressive nutrition essentially consists in the administration of high doses of amino acids throughout parenteral nutrition (PN) since the first days of life (DOL). A body of trials support the administration of high doses of parenteral amino acids in preterm newborns to improve nitrogen balance and guidelines for PN, published by the most important scientific societies, recommend to adopt this nutritional strategy to improve growth [3–5].

However, the optimal amount of calories associated with such a high amino acid supply has not yet been defined. The range of the protein:energy ratio, recommended by current guidelines, is very wide. Identifying the most appropriate energy supply based on protein intake is not easy. If a low caloric intake may impair growth, an energy overload may increase the risk of side effects associated with PN (i.e. hyperglycemia, hypertriglyceridemia, metabolic acidosis), particularly dangerous for critically ill individuals [1,6,7].

In light of these considerations, we aimed to investigate in a quasi-experimental study, the effects of different PN energy intakes, on postnatal growth of preterm newborns. An additional purpose of the study was to evaluate the safety of high caloric intake given in the first week of life.

## Methods

### Study design

We compared two Cohorts of newborns, which received different energy intakes through PN in the first weeks of life, in a quasi-experimental study design. Cohort A included newborns consecutively observed in NICU from 1^st^ January 2015 to 31 January 2016. After that period, PN protocol was changed as described in **Table 1**. From 1^st^ February 2016 to 31 March 2017, we enrolled newborns in the Cohort B. The two PN protocols adopted were different for energy intake, but not for protein intake of the first week of life (**Table 1**).

**Table 1:** Parenteral nutrition protocol of the study cohorts.

	Birth weight < 1000 g		Birth weight ≥ 1000 g	
	Cohort A	Cohort B	Cohort A	Cohort B
**Energy *(kcal/kg/day)***				
Starting dose	55	45	60	45
Target dose	120	105	110	100
**Proteins *(g/kg/day)***				
Starting dose	2.0	2.0	2.0	2.0
Target dose	4.0	4.0	3.5	3.5
**Dextrose *(g/kg/day)***				
Starting dose	7.0	7.0	8.5	7.0
Target dose	16.0	14.0	15.0	14.5
**Lipids *(g/kg/day)***				
Starting dose	2.0	1.0	2.0	1.0
Target dose	4.0	3.5	3.5	3.0

Starting dose was administered at the age of 0 days, Target dose was reached at the age of 7 days of life.

### Study population

We included all newborns with gestational age (GA) <32 weeks or body birth weight (BW) <1500 g, consecutively admitted to the NICU of Policlinico Umberto I, La Sapienza University of Rome. We excluded infants with major congenital malformations, inborn errors of metabolism, congenital infections, hospital discharge or death within 72 hours of life.

### Nutritional protocol

Human milk (HM) of own mother, without fortifications, was given fresh, as soon as possible after birth, if available. The preterm formula (PF) was administered to the infants when HM was not available or sufficient. Minimal enteral feeding started within 48 hours after birth at 10–20 ml/kg/day. The amount was increased by 20–30 ml/kg/day if enteral nutrition (EN) was tolerated. We withheld enteral feeding in case of severe abdominal distension, emesis, ileus with visible intestinal loops, blood in the stools, apnea, bradycardia, inadequate perfusion and hemodynamic instability [8]. No changes were made regarding enteral feeding policy during the two study periods.

The PN started immediately after birth in both study Cohorts. The PN was administered via central vascular access to maintain adequate fluids, electrolytes and nutrient intakes until exclusive enteral diet (120 kcal/kg/day) was achieved.

The overall fluid intake administered by combined EN and PN started with 70–90 ml/kg/day and increased by 10–20 ml/kg/day until the achievement of 150–180 ml/ kg/day, which was aimed to be reached by 7 to 10 DOL [1]. Preterm HM was assumed to contain 65 Kcal/100 ml (1.5 g of protein/100 ml, 3.5 g of fat/100 ml, 6.9 g of carbohydrate/100 ml). Macronutrient contents of formula (Pre-Nidina Nestlè ®, Milan, Italy) and of PN were calculated based on the published manufacturer’s labels and included proteins (TrophAmine® 6% Braun Medical Inc. Irvine, USA), lipids (Smoflipd ®, Fresenius Kabi, USA), and carbohydrates (Dextrose injection 10%, Fresenius Kabi, USA) expressed in g/kg/day. In PN, energy content was calculated by each nutrient as previously recommended [9].

### Outcomes

We considered as primary outcome the length at 24 months of corrected age. Secondary outcomes were the rate of patients with extrauterine growth retardation (EUGR) at 36 weeks of postmenstrual age (PMA), growth velocity, length of the hospital stay, survival, morbidity and PN associated complications, within 36 weeks of PMA, weight and head circumference at 12 and 24 months of life.

### Data collection

Medical staff was blinded to the study aims, but not to eligibility criteria. Researchers not involved in the clinical practice, provided information to the parents and collected all data useful for statistical analysis. A third party observer, not involved in the previous steps, was involved to collected data on primary and secondary outcomes. A blinded statistician performed data analysis.

We prospectively recorded prenatal, perinatal, and postnatal data in a specific data form. All infants were monitored until discharge, transfer to other hospital or death. The GA, BW, gender, type of delivery, antenatal steroid administration and occurrence of all relevant obstetric information were collected. Apgar score at 1^st^ and 5^th^ minute after birth, pH and base excess on cord blood, Clinical Risk Index for Babies (CRIB) II score, intensive care, need and duration of mechanical ventilation were prospectively recorded [10]. Data on PN, EN and feeding tolerance were collected daily. Data on daily cumulative parenteral and enteral nutritional intake were reported in a specific data form. We also collected data on length of the hospital stay, survival, morbidity and PN associated complications, within 36 weeks of PMA. Morbidity was defined as the presence of at least one of the major prematurity complications as necrotizing enterocolitis (NEC) Bell-Stage ≥ 2, intraventricular haemorrhage (IVH) stage≥ 2, periventricular leukomalacia (PVL), late-onset culture proven sepsis, retinopathy of prematurity (ROP) stage ≥ 3 and bronchopulmonary dysplasia (BPD). Diagnosis of NEC, BPD, IVH, PVL, ROP and late-onset culture proven sepsis were performed according with standard criteria, by physicians unaware of the study design and aims, as previously described [11,12].

In order to study the occurrence of PN related complications, we collected data on glycemia, calcemia, phosphatemia, azotemia, triglyceridemia and metabolic acidosis. Discharge from hospital was decided by the same physicians that evaluated eligibility, following standard criteria [13].

Nurses unaware of the study aims measured growth parameters. Weight, measured by digital scale, was recorded daily from birth to 36 weeks of PMA, at 12 and 24 months [14,15]. Length was measured from the top of the head to the sole of the feet, using a neonatal stadiometer at birth, 36 weeks of PMA and at 12 and 24 months [14,15]. Head circumference, evaluated by tape measurement, were collected at birth, 36 weeks of PMA and at 12 and 24 months [14,15]. Growth velocity during hospitalization was calculated as previously described [16]. We calculated weight, length and head circumference percentiles for sex and corrected age using on standard growth charts [14,15]. We defined EUGR as the reduction > 1SD (-1.28) in anthropometric parameters Z-Score between birth and 36 weeks of PMA [17]. We also collected data on health status of enrolled infants during the first 24 months of life (i.e. infections requiring hospitalization, atopic disease, asthma, major surgery, organ failure, chronic diseases) [18,19].

### Ethics

The study was conducted in conformity with World Medical Association Declaration of Helsinki for medical research involving human subjects, it was approved by Ethics Committee of Policlinico Umberto I, University La Sapienza of Rome (number 5089) and registered at the Australian New Zealand Clinical Trials Registry (ACTRN12619001773123, www.anzctr.org.au/ACTRN12619001773123.aspx). We obtained a written informed consent from all parents. Relevant summary-level statistics are presented in the manuscript and supplementary materials. Individual-level data cannot be shared publicly because of privacy laws (Italian Law: D.Lgs. n. 196/2003). Data are available from Department of Maternal and Child Health Policlinico Umberto I, University La Sapienza, Rome, Italy Institutional Data Access (contact via mail dipartimentouniversitario.misu@uniroma1.it) for researchers who meet the criteria for access to confidential data.

### Statistics

Statistical analysis was performed per protocol, using Statistical Package for Social Science software for Microsoft Windows (SPSS Inc, Chicago, IL), version 22.0. We checked for normality using Shapiro-Wilk test. The mean and 95% confidence interval summarised continuous variables. We compared the Cohorts using chi-square test for categorical variable and t-test or Mann-Whitney for paired and unpaired variables.

We applied linear regression analysis considering as dependent variable standardized and unstandardized weight and length measures 24 months of life and, as covariates, GA at birth, gender, age of the mother, morbidity, pH at birth and Cohort assignment or actual energy intake of the first weeks of life through PN. We chose the covariates that have been demonstrated to influence growth of infants born preterm in previous evidence [14,15,20]. The R-squared was used as a measure of model fit.

The level of significance for all statistical tests was 2-sided (p<0.05). On the basis of our preliminary data, we calculated for primary outcome a minimum sample size of 100 patients (2-sample t-test, 90% of power in hypothesis test, 0.05 of type 1 error, 2-tailed test, drop out 20%) to demonstrate a difference of 5 cm in length (85 vs. 80 cm, SD 7 cm) at 24 months of life. Secondary outcome sample power calculation indicated that a minimum sample size of 132 patients were required (chi-square, 80% of power in hypothesis test, 0.05 of type 1 error, 2-tailed test, drop-out 15%) to demonstrate a difference of 25% (20% vs. 45%) in EUGR rate.

## Results

### Study population

**Fig 1:**
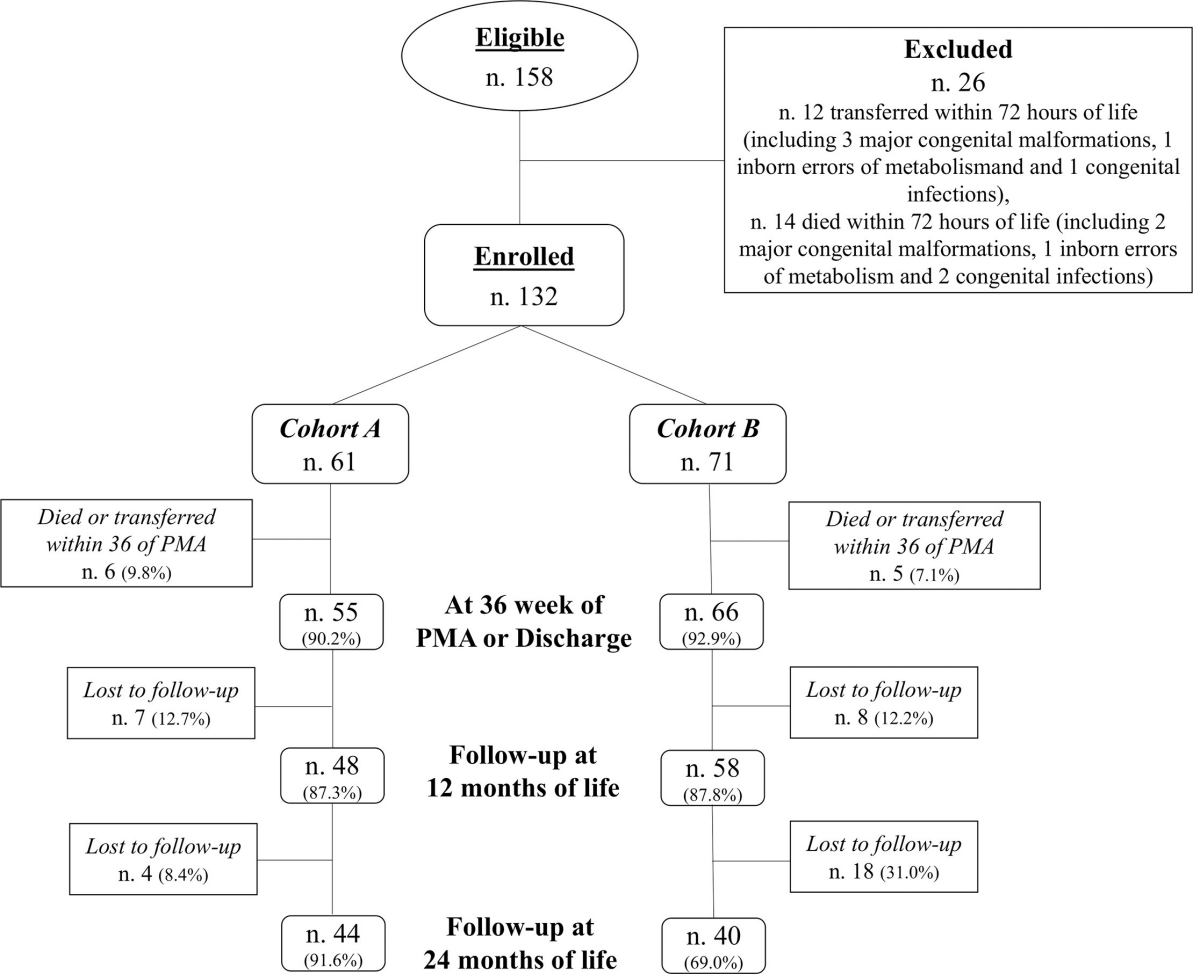
PMA: Postmenstrual age.

Of 158 eligible newborns, 132 were enrolled during the study period (**Fig 1**). Data regarding baseline clinical characteristics of enrolled newborns were reported in **Table 2.** The two Cohorts were similar for baseline characteristics (**Table 2**).

**Table 2:** Baseline characteristics of enrolled newborns.

	Cohort A *n = 61*	Cohort B *n = 71*	*p value*
**Gestational age, weeks**	29 (29 to 30)	30 (29 to 30)	0.322
**Birth weight, g**	1204 (1110 to 1299)	1236 (1153 to 1319)	0.618
**Male sex, No. (%)**	34 (55.7)	36 (50.7)	0.603
**Cesarean section, No. (%)**	54 (88.5)	59 (83.1)	0.460
**Caucasian, No. (%)**	53 (86.9)	62 (87.3)	1.000
**Unemployed Parents, No. (%)**	3 (4.9)	2 (2.8)	0.662
**Antenatal corticosteroids ^a^, No. (%)**	39 (63.9)	44 (62.0)	0.858
**IUGR, No (%)**	6 (9.8)	11 (15.5)	0.437
**Pregnancy-induced hypertension, No. (%)**	14 (23.0)	17 (23.9)	1.000
**SGA, No. (%)**	13 (21.3)	19 (26.8)	0.683
**Twins, No. (%)**	15 (24.6)	18 (25.4)	1.000
**1-min Apgar score**	5 (5 to 6)	5 (5 to 5)	0.762
**5-min Apgar score**	7 (7 to 8)	7 (7 to 8)	0.702
**pH at birth**	7.3 (7.2 to 7.3)	7.3 (7.2 to 7.3)	0.474
**Base excess on cord blood, mmol/L**	-6.2 (-7.3 to 5.1)	-5.2 (-6.1 to 4.4)	0.173
**CRIB II score ^b^**	6 (5 to 7)	6 (5 to 7)	0.639
**Non-invasive ventilation, No. (%)**	44 (74.6)	49 (69.0)	0.560
**PDA^c^, No. (%)**	21 (34.4)	19 (26.8)	0.350
**CVO (duration), days**	6 (5 to 7)	5 (5 to 6)	0.491
**PICC (duration), days**	11 (7 to 14)	8 (6 to 11)	0.373
**Inotrope, No. (%)**	9 (14.8)	6 (8.5)	0.283
**Age at start of EN, age in days**	3 (1 to 5)	3 (2 to 3)	0.488
**Start of EN before to 72h, No (%)**	44 (75.9)	56 (81.2)	0.518
**FEF, days after birth**	15 (12 to 18)	16 (12 to 21)	0.621
**Duration of PN, days**	14 (11 to 17)	12 (10 to 15)	0.418
**Energy intake 0–7 DOL through PN, kcal/Kg/day**	538.1 (502.9 to 573.0)	405.8 (367.6 to 444.0)	0.002
**Energy intake 0–14 DOL through PN, kcal/Kg/day**	843.1 (723.3 to 962.9)	621.1 (525.9 to 716.3)	0.004
**Protein Intake 0–7 DOL through PN, g/Kg/day**	22.3 (18.7 to25.8)	20.3 (14.3 to 26.3)	0.527
**Protein Intake 0–14 DOL through PN, g/Kg/day**	51.9 (38.8 to 65.1)	42.9 (15.5 to 70.3)	0.457

(a) Intramuscular steroid cycle in two doses of 12 mg over a 24-hour period

(b) CRIB II: clinical risk index for babies, without temperature measures

(c) PDA (Patent Ductus Arteriosus); EN: enteral nutrition; FEF: full enteral feeding; PN: Parenteral Nutrition; DOL: days of life. Data were expressed as mean (lower to upper limits 95% confidence interval), when not specified.

### Nutritional intake

**Fig 2:**
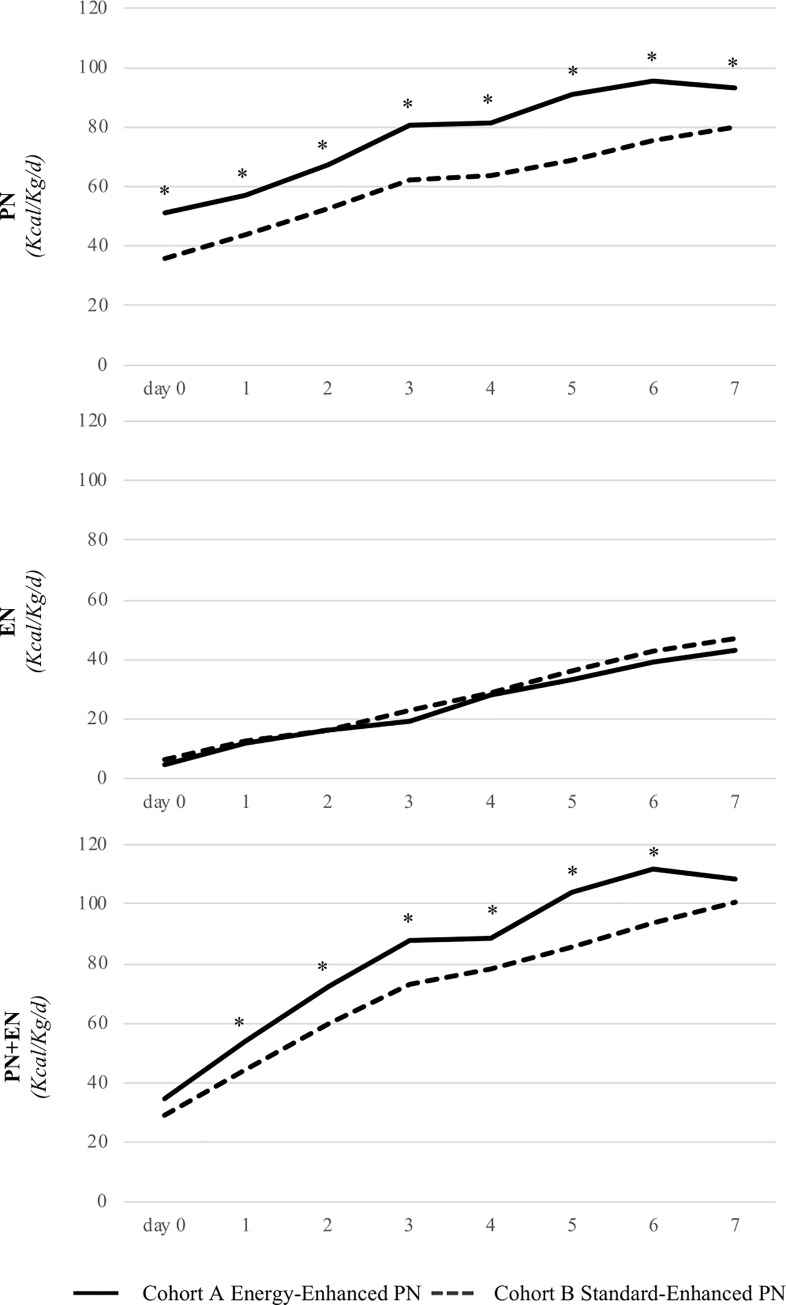
Energy intake included calories coming from protein; PN: parenteral nutrition; EN: enteral nutrition; * Cohort A vs Cohort B p<0.05.

In the **Table 2** we also showed that start of EN, tolerance, time to reach full enteral feeding (FEF) and protein intake were similar in the two study Cohorts. Whilst, they were different for the actual energy intake in the first week of life (**Table 2** and **Fig 2**).

### Outcomes

In the **Table 3** we report results regarding growth in the first 24 months of life of enrolled infants. Unstandardized body weight and length were increased in the Cohort A compared with Cohort B at 24 months of life (**Table 3**). No significant differences were observed in the Z-Score of the main long-term growth parameters between the two Cohorts (**Table 3**). In the **S1 Table** we showed results of sensitivity analysis performed on the newborns small for gestational age (SGA) at birth. In these newborns we observed that unstandardized, but not standardized, growth parameters were increased in Cohort A compared with Cohort B (**S1 Table**).

**Table 3:** Growth in the first 24 months of life of enrolled infants.

	Cohort A	Cohort B	*p value*
**At 12 months of life**	*n = 48*	*n = 58*	
**Body weight, g**	9015 (8660 to 9369)	8860 (8514 to 9205)	0.535
**Body weight *Z-Score***	-0.3 (-0.6 to 0)	-0.3 (-0.6 to 0)	0.892
**Head circumference, cm**	45.1 (44.7 to 45.6)	45.7 (44.9 to 46.5)	0.224
**Head circumference *Z-Score***	-0.3 (-0.6 to 0)	0.4 (-0.2 to 1)	0.059
**Length, cm**	74.4 (73.4 to 75.5)	72.9 (71.3 to 74.5)	0.128
**Length *Z-Score***	-1.3 (-0.5 to 0.2)	-0.4 (-1.1 to 0.3)	0.531
**BMI, Kg/m^2^**	16.2 (15.8 to 16.6)	16.9 (15.9 to 17.9)	0.232
**BMI, Kg/m^2^*Z-Score***	-0.4 (-0.7 to -0.1)	0 (-0.6 to 0.6)	0.277
**Weight for Length *Z-Score***	-0.4 (-0.7 to 0)	-0.2 (-0.5 to 0.1)	0.549
**At 24 months of life**	*n = 44*	*n = 40*	
**Body weight, g**	11136 (10656 to 11616)	10390 (9868 to 10912)	0.036
**Body weight *Z-Score***	-0.7 (-1.1 to -0.4)	-0.9 (-1.3 to -0.5)	0.613
**Head circumference, cm**	47.5 (47.0 to 48.1)	46.9 (46.3 to 47.4)	0.073
**Head circumference *Z-Score***	-0.3 (-0.7 to 0.1)	-0.4 (-0.7 to 0)	0.692
**Length, cm**	85.0 (83.8 to 86.2)	81.3 (79.7 to 82.8)	< 0.001
**Length *Z-Score***	-0.9 (-1.2 to -0.6)	-1.3 (-1.7 to -0.9)	0.175
**BMI, Kg/m^2^**	15.3 (14.9 to 15.7)	15.7 (15.1 to 16.2)	0.316
**BMI, Kg/m^2^*Z-Score***	-0.3 (-0.6 to 0)	-0.1(-0.5 to 0.2)	0.569
**Weight for Length *Z-Score***	-0.4 (-0.7 to -0.1)	-0.3 (-0.7 to 0)	0.798

Data were expressed as mean (lower to upper limits 95% confidence interval).

No significant difference was observed in the rate of newborns with EUGR in Cohort A (28%) compared with Cohort B (40%, p = 0.180). Daily growth velocity (g/Kg/d) and exponential growth velocity, from birth weight rescue to 36 weeks of PMA, were similar when we compared Cohort A vs. Cohort B (A 15.8, 95% CI 14.7 to 16.9 g/Kg/d vs. B 16.1, 95% CI 10.5 to 21.7 g/Kg/d, p = 0.914; A 16.3, 95% CI 15.1 to 17.4 g/Kg/d vs B 16.5, 95% CI 10.9 to 22.2 g/Kg/d, p = 0.925; respectively). Considering observed primary outcome values, we estimated a power of 82.4% through post-hoc sample size calculation (0.05 of type 1 error, 2-tailed test). Whereas, secondary outcome post-hoc sample size calculation showed a power of 30.1% (0.05 of type 1 error, 2-tailed test).

In the **Table 4** and **Table 5**, we report results of multivariate analysis. Linear regression analysis showed that only Cohort A assignment and only actual energy intake in the first week of life through PN were positively related with length at 24 months of life (**Table 4** and **Table 5**). The GA, sex, age of mother, morbidity and pH at birth were not related with dependent variables (**Table 4** and **Table 5**). Moreover, multivariate analysis showed a marginally insignificant association between Cohort A assignment and body weight at 24 months of life (**Table 4**).

**Table 4:** Linear regression analysis to evaluate the influence of covariates (gestational age, gender, pH at birth, age of mother, morbidity, Cohort assignment) on body weight and length at 24 months of life.

		B	Std Err.	β	p value	95.0% Confidence Interval	
						Lower Limit	Upper Limit
***Unstandardized Body weight (R*^*2*^*= 0*.*086 R*^*2*^*adj = 0*.*006)***	**Gestational Age**	-51.046	77.930	-0.082	0.515	-206.554	104.461
	**Sex**	540.799	392.237	0.165	0.172	-241.898	1323.496
	**Age of mother**	-14.814	32.608	-0.055	0.651	-79.883	50.256
	**Morbidity °**	-141.762	579.562	-0.030	0.808	-1298.261	1014.736
	**pH at birth**	982.867	2480.941	0.047	0.693	-3967.773	5933.507
	**Cohort**	742.968	388.047	0.227	0.060	-31.367	1517.303
***Unstandardized Length (R*^*2*^*= 0*.*255 R*^*2*^*adj = 0*.*189)***	**Gestational Age**	-0.118	0.203	-0.065	0.564	-0.524	0.288
	**Sex**	1.961	1.024	0.207	0.060	-0.082	4.004
	**Age of mother**	0.078	0.085	0.101	0.363	-0.092	0.248
	**Morbidity °**	-1.449	1.513	-0.108	0.342	-4.468	1.570
	**pH at birth**	8.448	6.476	0.139	0.196	-4.475	21.370
	**Cohort**	3.527	1.013	0.373	< 0.001	1.505	5.548
***Body weight Z-Score (R*^*2*^*= 0*.*012 R*^*2*^*adj = -0*.*075)***	**Gestational Age**	-0.023	0.057	-0.053	0.685	-0.137	0.091
	**Sex**	-0.077	0.288	-0.033	0.790	-0.651	0.497
	**Age of mother**	-0.014	0.024	-0.072	0.568	-0.061	0.034
	**Morbidity °**	-0.072	0.425	-0.022	0.866	-0.921	0.777
	**pH at birth**	-0.022	1.821	-0.001	0.991	-3.655	3.612
	**Cohort**	0.154	0.285	0.067	0.590	-0.414	0.722
***Length Z-Score (R*^*2*^*= 0*.*059 R*^*2*^*adj = -0*.*024)***	**Gestational Age**	-0.009	0.056	-0.019	0.879	-0.121	0.104
	**Sex**	0.096	0.284	0.041	0.736	-0.471	0.663
	**Age of mother**	0.017	0.024	0.087	0.485	-0.031	0.064
	**Morbidity °**	-0.380	0.420	-0.114	0.396	-1.217	0.458
	**pH at birth**	1.499	1.796	0.100	0.407	-2.086	5.083
	**Cohort**	0.320	0.281	0.137	0.259	-0.241	0.881

° NEC (Necrotizing Enterocolitis) or IVH (Intraventricular Hemorrhage) stage ≥ 4 or PLV (Periventricular Leucomalacia) or BPD (Bronchopulmonary Dysplasia) or late-onset culture proven Sepsis. R^2^ = R-squared; R^2^ adj = R-squared adjusted.

**Table 5:** Linear regression analysis to evaluate the influence of covariates (gestational age, sex, pH at birth, age of the mother, morbidity, energy intake of first week of life through parenteral nutrition) on body weight and length at 24 months of life.

		B	Std Err.	β	p value	95.0% Confidence Interval	
						Lower Limit	Upper Limit
***Unstandardized Body weight (R*^*2*^*= 0*.*045 R*^*2*^*adj = -0*.*039)***	**Gestational Age**	-40.313	83.818	-0.064	0.632	-207.558	126.933
	**Sex**	525.904	495.068	0.161	0.199	-282.395	1334.204
	**Age of mother**	-4.730	32.873	-0.018	0.886	-70.327	60.867
	**Morbidity °**	-133.848	592.761	-0.029	0.822	-1316.684	1048.987
	**pH at birth**	934.794	2553.310	0.045	0.715	-4160.256	6029.844
	**Energy intake 0–7 DOL through PN**	0.790	1.049	0.096	0.454	-1.303	2.882
***Unstandardized Length (R*^*2*^*= 0*.*179 R*^*2*^*adj = 0*.*107)***	**Gestational Age**	-0.007	0.225	-0.004	0.975	-0.455	0.441
	**Sex**	1.756	1.085	0.186	0.110	-0.410	3.922
	**Age of mother**	0.125	0.088	0.161	0.161	-0.051	0.301
	**Morbidity °**	-1.372	1.588	-0.102	0.391	-4.542	1.797
	**pH at birth**	8.975	6.842	0.148	0.194	-4.678	22.628
	**Energy intake 0–7 DOL through PN**	0.006	0.003	0.257	0.033	0.001	0.012
***Body weight Z-Score (R*^*2*^*= 0*.*012 R*^*2*^*adj = -0*.*075)***	**Gestational Age**	-0.036	0.060	-0.081	0.553	-0.156	0.084
	**Sex**	-0.047	0.291	-0.020	0.873	-0.627	0.533
	**Age of mother**	-0.011	0.024	-0.060	0.631	-0.058	0.036
	**Morbidity °**	-0.080	0.425	-0.024	0.852	-0.929	0.769
	**pH at birth**	-0.219	1.833	-0.015	0.905	-3.875	3.438
	**Energy intake 0–7 DOL through PN**	0.000	0.001	-0.073	0.576	-0.002	0.001
***Length Z-Score (R*^*2*^*= 0*.*042 R*^*2*^*adj = -0*.*043)***	**Gestational Age**	-0.010	0.060	-0.022	0.873	-0.129	0.110
	**Sex**	0.102	0.290	0.044	0.725	-0.476	0.680
	**Age of mother**	0.021	0.023	0.110	0.374	-0.026	0.068
	**Morbidity °**	-0.380	0.424	-0.115	0.373	-1.225	0.465
	**pH at birth**	1.407	1.825	0.094	0.443	-2.235	5.049
	**Energy intake 0–7 DOL through PN**	0.000	0.001	0.020	0.875	-0.001	0.002

° NEC (Necrotizing Enterocolitis) or IVH (Intraventricular Hemorrhage) stage ≥ 4 or PLV (Periventricular Leucomalacia) or BPD (Bronchopulmonary Dysplasia) or late-onset culture proven Sepsis; DOL: days of life. R^2^ = R-squared; R^2^ adj = R-squared adjusted.

We report in the **S2 Table** conditions influencing health status of the two Cohorts during the first 24 months of life. No difference was observed in the features characterizing health status of enrolled infants during the first 24 months of life (**S2 Table**). In the supplementary tables (**S3 Table** and **S4 Table**), we report characteristics of infants lost to follow up during the study. We did not find differences in baseline clinical findings of children lost to follow-up in the two Cohorts during the first 24 months of life (**S3 Table**). Children lost to follow-up were different for BW, rate of twins, Apgar score, pH and base excess on cord blood, and CRIB II score in comparison with those analysed at the end of follow-up period (**S4 Table**).

In the **Table 6,** we report side effects associated with the use of PN in enrolled newborns. The rate of PN associated complications were comparable between the two Cohorts (**Table 6**).

**Table 6:** Side effects associated with the use of parenteral nutrition in enrolled newborns.

			Cohort A *n = 61*	Cohort B *n = 71*	*p value*
**Glucose**	↑	*> 180 mg/dl*	23 (39.7)	27 (38.0)	0.850
	↓	*< 38 mg/dl*	3 (5.1)	8 (11.3)	0.173
**Calcium**	↑	*>2*.*4 mmol/l or > 11 mg/dl*	45 (76.3)	54 (76.1)	0.977
	↓	*< 1*.*6 mmol/l or < 7*.*5 mg/dl*	5 (8.5)	9 (12.7)	0.442
**Phosphorus**	↑	*> 3*.*1 mmol/l or > 9*.*6 mg/dl*	15 (25.4)	17 (23.9)	0.845
	↓	*<1*.*6 mmol/l or < 5 mg/dl*	16 (27.1)	21 (29.6)	0.757
**Urea**	↑	*> 5 mmol/l or > 14 mg/dl*	40 (67.8)	38 (53.5)	0.098
	↓	*< 2*.*9 mmol/l or < 8*.*1 mg/dl*	21 (35.6)	26 (36.6)	0.903
**Triglycerides**	↑	*> 150 mg/dl or > 1*.*8 mmol/l*	10 (32.3)	8 (28.6)	0.759
**Metabolic acidosis**	↑	*BE< 10 mmol or pH < 7*.*25 with pCO2 > 50*	13 (22.8)	17 (23.9)	0.880
**Overall Side effects**			58 (95.1)	70 (98.6)	0.255

Data were expressed as No (%).

In the **S5 Table**, we report the morbidity observed in the two study Cohorts. Morbidly was similar between the two Cohorts of the study (**S5 Table**). Length of hospital stay (A 61, 95% CI 57 to 77 days vs. B 61, 95% CI 59 to 76 days, p = 0.982), were comparable between the two Cohorts.

## Discussion

This study shows that the energy-enhanced PN in the first week of life has no significant effect on long-term growth. Although, we observed an improvement in unstandardized measures of body weight and length, no difference in body weight and length Z-Score was observed between the study Cohorts in the first 24 months of life. The increased energy supply in PN in the first week of life did not influence the occurrence of EUGR, length of hospital stay, morbidity or the occurrence of PN associated complications.

Previous studies on enhanced PN protocols in preterm infants have mainly focused on amino acid intake. There is a small evidence available on long-term effects of different PN energy supplies in preterm infants [21]. Comparative trials have not clarified the role of energy intake role in comparison with protein intake. In intervention trials, an increase in energy intake has always been associated with an increased protein intake. Furthermore, actual macronutrients intake has seldom been evaluated. The RCTs demonstrated a better brief-term growth in newborns receiving a high energy intake, but only when associated with a high amino acid intake [22–24]. Poindexter et al. also found an improvement in growth at 18 months in male infants who received high energy and protein doses through PN early in the life [23]. Nevertheless, none of these studies showed whether the effects observed on growth were due to protein or energy intake [25–27]. Unlike previous trials, our study was designed to assess the effects on long-term growth of two PN protocols, different for energy intakes but similar for protein intakes. In addition, we performed comparison analysis after actual nutrient intake control. We verified that energy amount actually differed while having similar protein intake in the two cohorts. Therefore, our results suggest that possible energy intake effect on growth may be independent from protein intake.

Evidence from RCTs demonstrated that energy intake in the first weeks of life does not significantly influence morbidity [23,24,28,29]. Previous studies in children and adults admitted in intensive care unit showed an increased morbidity associated with enhanced PN [30,31]. Recent studies reported an increased risk of morbidity (i.e. NEC and ROP), but a reduction of PLV in newborns receiving an enhanced PN in early life [6,26]. These studies adopted PN protocols both enhanced for protein and energy intake, whereas, our study, focused only on PN energy intake, suggests that caloric amounts did not contribute to increase the morbidity in preterm newborns. Further studies are needed to clarify this relationship. Despite using recommended doses during the entire study period, a high rate of metabolic complications frequency was noted in both study Cohorts [3,4]. This poses concerns regarding the safety of recommended energy intake in very preterm newborns. We believe that it is unacceptable to tolerate that up to 98% of newborns receiving PN may develop a related complication.

Our results should be balanced with several study limitations. The association between early energy intake and growth at 24 months of life may be related to the effects of chance (random error), bias or confounding factors.

The two Cohorts were similar for conditions affecting growth in the first 24 months. We verified that effect on long-term growth of energy-enhanced PN protocol persisted even after correction for confounding variables. Despite of everything, other confounding variables, unknow or not considered in our statistical analysis, may have influenced the study results. R-squared observed in our model suggested that other covariates not included in the model may have influenced the results of multivariate analysis. Growth in the first two years of life depend on a number of factors. In this model, we considered some conditions that could affect infant growth. Previous studies in similar setting do not report R-squared values [21,26–29]. Bonsante et al. reported R-squared value of their linear regression analysis [7]. They only studied the influence of confounding variables on metabolic acidosis but not on growth parameters. Further studies are necessary to better address this issue.

This is not a randomized trial. Individualized PN solutions corrections are the milestone of our policy on PN, in order to avoid deleterious consequences of complications related with the intravenous administration of dextrose or lipid emulsions. Physicians prescribing PN were not blinded. Despite being a potential information bias, we have preferred that neonatologists taking care of the babies knew the composition of PN, in order to make immediate corrections in the case of hyperglycaemia or hypoglycaemia. To limit selection bias, physicians evaluating eligibility were blinded to the study aims and used objective inclusion criteria (such as GA, BW). To limit observer bias, the data for the analysis were collected by researchers not involved in eligibility assessment and who were unaware of cohort assignment. We discussed and defined a protocol for the collection, measurement and interpretation of data before starting the study. A third party observer was involved to collect data on primary and secondary outcomes. Finally, a blinded statistician performed the data analysis.

We divided the Cohorts on a temporal basis. This has increased the risk of bias. Despite no changes in the policies care during the entire study period and similar baseline characteristics of the two Cohorts, it is not possible to exclude that unknown differences in the clinical practice or changes in the medical staff composition, may have influenced the results.

We observed a statistically significant effect of a nutritional intervention performed in the first week of life at 24 months of life, but not at 36 weeks of PMA or at 12 months of life. We hypothesized different explanations for this phenomenon. We designed the study to demonstrate a difference in length at 24 months. A sample size calculation was made on the primary outcome. The study is not powered to demonstrate differences in unstandardized growth parameters at previous time points and in standardized growth parameters at 24 months of life. Probably, also for this reason, differences in unstandardized growth parameters were not statistically significant at time points before that of 24 months and standardized growth parameters were not statistically significant at 24 months of life. Furthermore, loss to follow-up higher in the Cohort B compared with Cohort A, may explain, at least in part, the different growth pattern observed in the two study Cohorts.

Nevertheless, as others have previously suggested, we speculate that an epigenetic effect produced by a nutritional intervention performed in a critical window, may be visible later, without effects in the first period of life [19,32].

In conclusion, an energy-enhanced PN protocol is not associated with long-term growth improvement. The optimal PN should promote growth and at the same time limit any harmful effects in this particular population. The high prevalence of PN-associated complications observed in both Cohorts of the study raises concerns about the implementation of current recommendations [3,4]. More caution might be necessary for aggressive nutritional strategies in the early stages of life for preterm infants. Further studies are critically needed to ascertain the safety of increased energy intake through PN in the first week of life.

## Supporting information

S1 Table Sensitivity analysis performed on SGA. (DOCX)

S2 Table Clinical characteristics of enrolled children during the first 24 months of life. (DOCX)

S3 Table Baseline clinical findings of children lost to the follow-up during the first 24 months of life. (DOCX)

S4 Table Baseline characteristics of children lost to follow-up compared with infants analyzed at 24 months of life. (DOCX)

S5 Table Morbidity of enrolled children. (DOCX)
