# Supplementary material for: Long-term effects on growth of an energy-enhanced parenteral nutrition in preterm newborn: A quasi-experimental study
Source: PLoS One. 2020 Jul 6;15(7):e0235540. doi: 10.1371/journal.pone.0235540 (PMC7337335; doi:10.1371/journal.pone.0235540)
Supplement: S1 Table — (DOCX) [file pone.0235540.s001.docx]

**Table S1. Sensitivity analysis performed on SGA.**

|  | Cohort A | Cohort B |
| --- | --- | --- |
| SGA | *n=9* | *n=9* |
| Body weight at 24 months, g | 10123 (9328 to 10919)* | 9089 (8512 to 9665) |
| Body weight at 24 months, *Z-Score* | -1.6 (-2.3 to -0.9) | -2.0 (-2.5 to -1.4) |
| Head circumference at 24 months, cm | 46.7 (45.4 to 48.1) | 46.1 (44.6 to 47.6) |
| Head circumference at 24 months, *Z-Score* | -1.0 (-2.0 to 0) | -1.0 (-1.9 to 0) |
| Length at 24 months, cm | 82.6 (79.7 to 85.4)* | 78.5 (76.2 to 80.8) |
| Length at 24 months, *Z-Score* | -1.8 (-2.4 to -1.1) | -2.2 (-2.6 to -1.8) |
| BMI at 24 months, Kg/m^2^ | 14.8 (14.0 to 15.7) | 14.8 (13.8 to 15.7) |
| BMI at 24 months *Z-Score* | -0.7 (-1.5 to 0) | -0.8 (-1.6 to -0.1) |
| Weight for Length at 24 months, *Z-Score* | -1.0 (-1.7 to -0.2) | -1.2 (-1.8 to -0.5) |

Notes. * vs Cohort B, p < 0.05; Data were expressed as mean (lower to upper limits 95% confidence interval).
