# Supplementary material for: Long-term effects on growth of an energy-enhanced parenteral nutrition in preterm newborn: A quasi-experimental study
Source: PLoS One. 2020 Jul 6;15(7):e0235540. doi: 10.1371/journal.pone.0235540 (PMC7337335; doi:10.1371/journal.pone.0235540)
Supplement: S2 Table — (DOCX) [file pone.0235540.s002.docx]

**Table S2. Clinical characteristics of enrolled children during the first 24 months of life.**

|  | Cohort A  *n=44* | Cohort B  *n=40* |
| --- | --- | --- |
| Infection requiring hospitalization | 8 (18.2) | 9 (22.5) |
| Atopic diseases | 3 (6.8) | 3 (7.5) |
| Asthma | 1 (2.3) | 5 (12.5) |
| Major surgery | 0 (0) | 1 (2.5) |
| Organ failure | 1 (2.3) | 0 (0) |
| Chronic diseases | 1 (2.3) | 2 (5.0) |

Notes. Data were expressed as No. (%).
