# Supplementary material for: Long-term effects on growth of an energy-enhanced parenteral nutrition in preterm newborn: A quasi-experimental study
Source: PLoS One. 2020 Jul 6;15(7):e0235540. doi: 10.1371/journal.pone.0235540 (PMC7337335; doi:10.1371/journal.pone.0235540)
Supplement: S3 Table — (DOCX) [file pone.0235540.s003.docx]

**Table S3. Baseline clinical findings of children lost to the follow-up during the first 24 months of life.**

|  | Cohort A  *n=17* | Cohort B  *n=31* |
| --- | --- | --- |
| Gestational age, weeks | 29 (28 to 30) | 30 (28 to 31) |
| Birth weight, g | 1069 (891 to 1247) | 1176 (1072 to 1281) |
| Male sex, No. (%) | 7 (41.2) | 15 (48.4) |
| Cesarean section, No. (%) | 16 (94.1) | 26 (83.9) |
| Caucasian, No. (%) | 14 (82.4) | 26 (83.9) |
| Antenatal corticosteroids ^a^, No. (%) | 10 (58.8) | 20 (64.5) |
| IUGR, No (%) | 3 (17.6) | 6 (19.4) |
| SGA, No. (%) | 4 (23.5) | 10 (32.3) |
| Twins, No. (%) | 1 (5.9) | 1 (6.1) |
| 1-min Apgar score | 5 (3 to 6) | 5 (4 to 5) |
| 5-min Apgar score | 7 (6 to 8) | 7 (7 to 8) |
| pH at birth | 7.2 (7.2 to 7.3) | 7.3 (7.2 to 7.3) |
| CRIB II score ^b^ | 8 (6 to 10) | 7 (6 to 9) |
| Age at start of EN, age in days | 2 (1 to 3) | 3 (1 to 4) |
| Start of EN before to 72h, No (%) | 13 (92.9) | 24 (82.8) |
| FEF, days after birth | 12 (5 to 18) | 13 (9 to 17) |
| Duration of PN, *days* | 12 (5 to 18) | 11 (8 to 13) |
| Body weight at 36 of PMA, g | 2052 (1802 to 2302) | 2203 (1960 to 2446) |
| Head circumference at 36 of PMA, cm | 30.1 (26.5 to 33.7) | 32.4 (30.7 to 34.1) |
| Length at 36 of PMA, cm | 44.6 (43.0 to 46.1) | 45.4 (43.6 to 47.2) |

Notes. (a) Intramuscular steroid cycle in two doses of 12 mg over a 24-hour period (b) CRIB II: clinical risk index for babies, without temperature measures; PMA: postmenstrual age; EN: enteral nutrition; FEF: full enteral feeding; PN: Parenteral Nutrition. Data were expressed as mean (lower to upper limits 95% confidence interval), when not specified.
