# Supplementary material for: Long-term effects on growth of an energy-enhanced parenteral nutrition in preterm newborn: A quasi-experimental study
Source: PLoS One. 2020 Jul 6;15(7):e0235540. doi: 10.1371/journal.pone.0235540 (PMC7337335; doi:10.1371/journal.pone.0235540)
Supplement: S4 Table — (DOCX) [file pone.0235540.s004.docx]

**Table S4. Baseline characteristics of children lost to follow-up compared with infants analyzed at 24 months of life.**

|  | Follow-up at 24 months  *n=84* | Lost to follow-up  *n=48* |
| --- | --- | --- |
| Gestational age, weeks | 30 (29 to 30) | 29 (28 to 30) |
| Birth weight, g | 1269 (1187 to 1350)* | 1138 (1049 to 1228) |
| Male sex, No. (%) | 48 (68.6) | 22 (45.8) |
| Cesarean section, No. (%) | 71 (84.5) | 42 (87.5) |
| Caucasian, No. (%) | 9 (10.7) | 8 (16.7) |
| Antenatal corticosteroids ^a^, No. (%) | 53 (63.1) | 30 (62.5) |
| IUGR, No (%) | 8 (9.5) | 9 (18.8) |
| SGA, No. (%) | 18 (21.4) | 14 (31.1) |
| Twins, No. (%) | 27 (32.1)* | 6 (12.5) |
| 1-min Apgar score | 5 (5 to 6)* | 5 (4 to 5) |
| 5-min Apgar score | 8 (7 to 8) | 7 (7 to 8) |
| pH at birth | 7.3 (7.3 to 7.3)* | 7.2 (7.2 to 7.3) |
| Base excess on cord blood, mmol/L | -5.1 (-5.8 to -4.3)* | -6.8 (-8.1 to -5.5) |
| CRIB II score ^b^ | 6 (5 to 6)* | 7 (6 to 8) |
| Age at start of EN, age in days | 3 (2 to 5) | 2 (1 to 3) |
| Start of EN before to 72h, No (%) | 63 (75.0) | 37 (86.0) |
| FEF, days after birth | 17 (14 to 21) | 13 (10 to 16) |
| Duration of PN, days | 14 (12 to 17) | 11 (8 to 14) |
| Body weight at 36 of PMA, g | 2307 (2190 to 2424) | 2141 (1980 to 2302) |
| Head circumference at 36 of PMA, cm | 33.1 (32.3 to 33.9) | 31.4 (29.8 to 33.1) |
| Length at 36 of PMA, cm | 45.9 (45.0 to 46.8) | 45.1 (43.9 to 46.2) |

Notes. (a) Intramuscular steroid cycle in two doses of 12 mg over a 24-hour period (b) CRIB II: clinical risk index for babies, without temperature measures; PMA: postmenstrual age; EN: enteral nutrition; FEF: full enteral feeding; PN: Parenteral Nutrition. * vs Cohort B, p < 0.05; Data were expressed as mean (lower to upper limits 95% confidence interval), when not specified.
