# Supplementary material for: Long-term effects on growth of an energy-enhanced parenteral nutrition in preterm newborn: A quasi-experimental study
Source: PLoS One. 2020 Jul 6;15(7):e0235540. doi: 10.1371/journal.pone.0235540 (PMC7337335; doi:10.1371/journal.pone.0235540)
Supplement: S5 Table — (DOCX) [file pone.0235540.s005.docx]

**Table S5. Morbidity of enrolled children.**

|  | Cohort A  *n=61* | Cohort B  *n=71* |
| --- | --- | --- |
| NEC | 2 (3.3) | 0 (0.0) |
| IVH | 2 (3.3) | 5 (7.0) |
| PLV | 2 (3.3) | 1(1.4) |
| Sepsis | 4 (6.6) | 5 (7.0) |
| ROP (stage ≥ 3) | 3 (4.9) | 6 (8.5) |
| BPD | 4 (6.6) | 5 (7.0) |
| Overall Morbidity | 27 (44.3) | 28 (39.4) |

Notes. NEC (Necrotizing Enterocolitis); IVH (Intraventricular Hemorrhage); PLV (Periventricular Leucomalacia); ROP (Retinopathy of Prematurity); BPD (Bronchopulmonary Dysplasia). Data were expressed as No. (%)
